# An Analytical Study on an Orthodontic Index: Index of Complexity, Outcome and Need (ICON)

**Published:** 2015-09

**Authors:** Sepide Torkan, Hamid Reza Pakshir, Hamid Reza Fattahi, Morteza Oshagh, Shahla Momeni Danaei, Parisa Salehi, Zohreh Hedayati

**Affiliations:** 1 Shiraz Orthodontic Research Center, Shiraz University of Medical Sciences, Shiraz, Iran.; 2 Shiraz Orthodontic Research Center, Dept. of Orthodontics, School of Dentistry, Shiraz University of Medical Sciences, Shiraz, Iran.

**Keywords:** ICON index, Orthodontics, Epidemiologic index, Validity, IOTN index

## Abstract

**Statement of the Problem:**

The validity of the Index of Complexity, Outcome and Need (ICON) which is an orthodontic index developed and introduced in 2000 should be studied in different ethnic groups.

**Purpose:**

The aim of this study was to perform an analysis on the ICON and to verify whether this index is valid for assessing both the need and complexity of orthodontic treatment in Iran.

**Materials and Method:**

Five orthodontists were asked to score pre-treatment diagnostic records of 100 patients with a uniform distribution of different types of malocclusions determined by Dental Health Component of the Index of Treatment Need. A calibrated examiner also assessed the need for orthodontic treatment and complexity of the cases based on the ICON index as well as the Index of Orthodontic Treatment Need (IOTN). 10 days later, 25% of the cases were re-scored by the panel of experts and the calibrated orthodontist.

**Results:**

The weighted kappa revealed the inter-examiner reliability of the experts to be 0.63 and 0.51 for the need and complexity components, respectively. ROC curve was used to assess the validity of the index. A new cut-off point was adjusted at 35 in lieu of 43 as the suggested cut-off point. This cut-off point showed the highest level of sensitivity and specificity in our society for orthodontic treatment need (0.77 and 0.78, respectively), but it failed to define definite ranges for the complexity of treatment.

**Conclusion:**

ICON is a valid index in assessing the need for treatment in Iran when the cut-off point is adjusted to 35. As for complexity of treatment, the index is not validated for our society. It seems that ICON is a well-suited substitute for the IOTN index.

## Introduction

The demand for orthodontic treatment has increased over the last decade in Iran as well as other countries along with an increase in general awareness of esthetics.[1] Policy making for orthodontic treatment and designating human and financial resources is only possible when accurate epidemiologic studies are carried out in the society and treatment needs are well clarified.

Although there is not a universally accepted measure for assessment of orthodontic treatment need,[2] different indices have developed over the years for an objective measurement of the need for orthodontic treatment. Recently, a new index has been developed that assesses the need for treatment, treatment complexity and outcome and is based on the general consensus of 97 orthodontists across the globe which is called the Index of Complexity, Outcome and Need (ICON).[3] It has been developed by Daniels and Richmond[3] in 2000 and is claimed to be simpler to assess than previously introduced indices. Since this index has been invented, its reliability and validity has been assessed in some ethnic groups [1, 4-7] and is yet to be evaluated in other racial groups. The cut-off value originally assigned by Daniels and Richmond was concluded not to be appropriate in a Dutch population and a higher value was suggested.[4] But it seemed to be reliable when assessed by calibrated orthodontists.[4] This index consists of five weighted measurements as the Aesthetic Component (AC) of the Index of Orthodontic Treatment Need (IOTN), upper arch crowding/spacing, the presence of crossbite, anterior vertical relationship (deep bite and open bite), and buccal segment interdigitation as suggested in Peer Assessment Rating (PAR) index.[8]

Even though the need for orthodontic treatment has been evaluated using the ICON score in some ethnic groups,[4-7] the importance of validating this index is still in debate before it can be employed as an extensive epidemiologic assessment tool in Iran. A new diagnostic method can be validated when it is compared with the gold standard which is the common sense of orthodontists in the case.[4-6]

The aims of this study were to assess the validity of the need for orthodontic treatment and complexity of treatment in the ICON index and to compare the level of agreement between the IOTN and ICON indices. 

## Materials and Method

In order to select the study sample, orthodontic diagnostic records of 650 patients at the Orthodontic Department, Shiraz University of Medical Sciences Shiraz Dental School were collected. The Dental Health Component (DHC) of the IOTN index of all the samples was measured and eventually 100 cases were selected that represented different types of malocclusion. Therefore, no randomization was carried out; instead a uniform study sample was provided. The selection was conducted to eventually have equal cases of different classes of malocclusion.

Panoramic radiographs, lateral cephalograms, and extraoral photographs as well as the study casts of the patients were collected as the diagnostic records. Panoramic radiograph were used to determine impactions, missing or blocked out teeth. All the records were either photographed or scanned and then imported into the PowerPoint software and consecutive slides were made from the diagnostic records of each patient.

Five volunteer orthodontists were invited to score the cases. The inclusion criteria for the expert panel were a minimum experience of 5 years in clinical practice and willingness to take part in the study.[1, 4-6] Before the session started, a calibrated orthodontist gave a brief detail regarding the ICON index. The examiners were then asked to fill out a form about each patient as the slides bearing their records were displayed. Each form was divided into two sections. The first part assessed the need for orthodontic treatment. All 100 cases were rated on a scale from 1 to 5; 1 indicating no need for treatment or a minimal need and 5 indicating a very high need based on their individual viewpoint. The orthodontists were asked to score each patient assuming a full compliance of the patient with no financial complications. The second part assessed the complexity of treatment of the cases and was ranked from 1 to 5 ranging from easy to very difficult. The orthodontists were invited not to talk or seek each other’s opinions during the session. There was no time limit in filling out the form but each orthodontist was asked to fill the form in a single session. The resulting scores obtained from the orthodontists were called the ‘clinical sense’. The participants were then asked to indicate which score they believe could represent the cut-off point above which the orthodontic treatment was definitely required for each patient. This was called the Indicated Treatment Point (ITP).[4]

The view of the majority of the raters determined the gold standard. Therefore, if more than 3 raters gave a certain score to a patient, the gold standard would be set to that number, providing the most popular opinion.

10 days later, 30 of the casts were randomly selected from the study sample to evaluate intra-examiner reliability and were displayed once more for the orthodontists to be scored via the same method. 

One calibrated orthodontist (Torkan)evaluated and scored the ICON index as well as the IOTN index for each case.[1, 5-6] After a month, 30 of these casts were re-scored by the same calibrated orthodontist. A table demonstrating different components assessed in the ICON index are presented in the Appendix 1.

All the obtained data was imported into the SPSS software version 9.1 (IBM Corporation). Simple kappa and weighted kappa tests were used to assess inter-examiner and intra-examiner reliability via the WinPepi software version 3.8.[9] ROC curve was used to evaluate the validity of the ICON index by moving the boundaries to find the best threshold in our society.

## Results

The IOTN was measured in all the cases (n= 100) prior to assessment by the panel in order to ensure a wide variety of cases based on the severity of malocclusions were included in the sample. Distribution of different types of malocclusion based on DHC is outlined in Table 1.

**Table 1:** IOTN: Index for Treatment Need

**** **IOTN grade**	**Treatment need**	**Frequency (%)**
1	No need	24
2	Little need	21
3	Borderline need	23
4	Great need	23
5	Very great need	8
Total cases		100

The mean ITP obtained from the 5 examiners was 3.1. Regression test was used to assess the intra-examiner reliability. Regarding the need for treatment, reliability was present in 5 orthodontists. Inter-examiner reliability of the panel of experts is summarized in Table 2. The weighted kappa revealed the inter-examiner reliability of the experts to be 0.63 and 0.51 for the need and complexity components, respectively.

**Table 2:** Inter-examiner reliability of the “gold standard” for need and complexity components (weighted Kappa test)

**Component **	**Inter-examiner reliability**	**P value**
need	0.63	*p*< 0.00
complexity	0.51	*p*< 0.00

**Figure 1:**
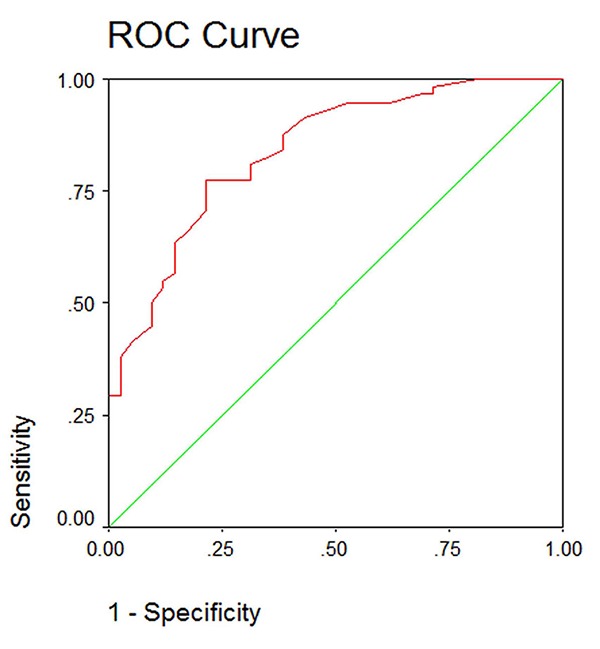
Sensitivity and specificity at different cut-off points for the panel of experts

When the intra-examiner reliability of the calibrated orthodontist was assessed, the kappa coefficient showed almost perfect agreement (kappa= 0.82, *p*< 0.00). After dichotomizing the results as “no treatment need” and “treatment required”, the intra-examiner reliability for the calibrated examiner was still “almost perfect” (kappa=0.89, *p*< 0.00).In order to evaluate the validity of ICON index in assessing the need for treatment, the sensitivity and specificity of ICON index at different cuff-off points was evaluated using the ROC curve. It was shown that the best cut-off point in our community for definite treatment need was 35.5 in lieu of 43 (Figure 1 and Table 3). A new cut-off point was adjusted at 35 in lieu of 43 as the suggested cut-off point. It showed the highest level of sensitivity and specificity in our society (0.77 and 0.78, respectively), but it failed to define definite ranges for the complexity of treatment.

**Table 3:** Different cut-off points with their sensitivity and specificity

**Cut-off**	**Sensitivity**	**1-specificity**
31.5	0.810	0.310
32.5	0.793	0.310
33	0.786	0.310
34.5	0.776	0.238
35	0.776	0.214
35.6	.0.724	0.214
37.5	0.707	0.214
39.5	0.655	.167
41.5	0.638	0.143
43	0.621	0.143
44	0.569	0.143
46.5	0.552	0.119
48.5	0.534	0.119

ROC curve was used to assess the thresholds suggested by Daniels and Richmond for treatment complexity. Table 4 compares the suggested threshold for the complexity of treatment and those obtained from our study.

**Table 4:** Thresholds for different degrees of complexity as suggested by ICON and the raters

ICON: Index of Complexity, Outcome and Need		
**Degree of complexity**	**Gold standard**	**ICON**
Easy	<8.5	<29
Mild	8.5-26.5	29-50
Moderate	27-39.5	51-63
Difficult	_	64-77
Very difficult	>40	>77

**Appendix 1:** Index of Complexity, Outcome and Need (ICON)

			**Score**				
**Components**	**0**	**1**	**2**	**3**	**4**	**5**	**Weight **
Aesthetic assessment (AC)		Score 1 to 10					5
Upper arch crowding	<2mm	2.1 to 5 mm	5.1 to 9mm	9.1 to 13mm	13.1 to 17mm	>17mm	5
Upper spacing	<2mm	2.1 to 5mm	5.1 to 9mm	>9mm		Impacted tooth	
Cross bite	No cross bite	Cross bite present					5
Incisor open bite	Edge to edge	<1mm	1.1 to 2mm	2.1 to 4mm	>4mm		4
Incisor overbite	<1/3 lower incisor coverage	1/3 to 2/3 coverage	2.3 up to fully covered	Fully covered			4
Buccal segment anterior-posterior	Cusp to embrasure only Class I.II or III	Any cusp relation up to but not including cusp to cusp	Cusp to cusp				3

## Discussion

The present study assessed the validity of an orthodontic index namely ICON among an Iranian population, with six experts as the gold standard. A clear investigation on the applicability and reliability of this index in Iran is required to help orthodontists decide about the need for orthodontic treatment especially in policy making on a national level. Also, with an increasing tendency amongst general practitioners in Iran to do orthodontic treatment for their patients, if this index represents a clear cut-off regarding the level of the complexity of the cases in Iran, it can help them better decide whether they have the knowledge or capability to proceed with the orthodontic treatment. ICON index was initially developed due to the shortcomings in other existing indices such as IOTN and PAR index.[3] This index assesses the need for orthodontic treatment, the complexity of the cases and the outcome of orthodontic treatment based on some tangible and easily measurable factors such as the presence of open bite, cross bite, upper arch crowding and spacing (appendix 1). The IOTN and PAR index have only been validated in United Kingdom and therefore, it is only fair to consider that they might not fully represent the opinion of international orthodontists.[10-11] Borzabadi-Farahani *et al.* concluded that the presence of an index to assess the complexity of treatment is of great importance since it provides a perspective of the type of treatment that should be expected for the patient as well the possible duration of treatment,[11] however, they did not assess whether this index was used by the proper cut-off point in Iran regarding the need for orthodontic treatment or the complexity of the cases. ICON is the only index that is designed to assess the need, complexity, degree of improvement and outcome of orthodontic treatment.[3] Even the PAR index only assesses the degree of deviation from normal occlusion and not the complexity of treatment.[8]

In order for the sample to represent all different types of malocclusions, we measured the distribution of DHC component of IOTN. The same method was carried out in a couple of previous studies;[4, 6] however, Savastano *et al.* could not provide an even distribution of different malocclusions.[5] It seems that challenging the examiners with different types of malocclusions can better assess the validity and usefulness of the ICON index in conventional practice.

The intra-examiner reliability of the experts was reported to be acceptable when each examiner re-scored 25% of the samples after 10 days. The inter-examiner reliability for the need and complexity components of the index was 0.63 and 0.51, respectively.

Based on the classification provided by Landis and Koch, the kappa in the range of 0.41 to 0.60 is considered as moderate agreement and 0.6 to 0.80 is considered as substantial agreement.[12] These results were in consistence with the results of Savastano *et al.* who reported the inter-examiner reliability to be 0.50 for complexity and 0.18 for outcome[5] in orthodontic treatment. However, in earlier studies, the level of agreement among practitioners was higher than our study. Firestone *et al.*[6] and Louwerse *et al.*[4] reported an inter-examiner agreement of 0.9 for the need component which was also in the same level of agreement as some other previous studies that evaluated the validity of other indices.[13-15] Most of these studies have used a local panel of orthodontists to obtain the gold standard,[4, 6] while in our study each orthodontist was graduated from different universities across the country. This might have affected their judgment on treatment need or complexity. This dispute, on the other hand, provides more freedom to generalize the results nationwide. It might also be attributed to the number of examiners, how old they have been and how much experience they have had in orthodontic practice. It can be speculated that if the number of examiner had been larger, higher levels of agreement could have been achieved.

The method through which the data were obtained from the examiners was also different in our study. In the present study, the complete orthodontic diagnostic records of each patient were provided for the raters similar to the method employed by Louwerse *et al.*;[4] however, in other studies only the dental casts were provided for evaluation.[3, 8, 15] It has been proposed that the complete diagnostic records of each patient (the lateral cephalogram, photographs, panoramic radiographs, and the study casts) which are used on a routine treatment planning in an orthodontic office can affect the external validity of the results.[4]

Regarding the validity of the ICON index, it has to be emphasized that this index has been validated against the opinion of 97 orthodontists across Europe and the United States and therefore is expected to show the highest level of sensitivity and specificity in that population. Once this index was developed, the sensitivity and specificity of this index was suggested to be 85.2 and 86.4, respectively.[3] Other studies reported almost the same percentages for sensitivity and specificity of the index at the aforementioned cut-off point.[4] In our country, ROC curve was used to assess the sensitivity and specificity of the cut-off point of 43. The sensitivity was 0.62 and specificity was 0.86. When the cut-off point was adjusted to 35, the highest level of sensitivity and specificity was reported to be 0.77 and 0.78, respectively. At this adjusted cut-off point the positive predictive value and negative predictive value were 88.2% and 58.3%, respectively. The overall accuracy of the index for orthodontic treatment need was 75.8%. The difference, apart from the fact that this index has not been validated in Iran, can be attributed to the difference in the insurance policy in different countries. It has been stated that the National Health Service (NHS) in the United Kingdom provides funding for patients with a DHC component of 3 and an AC of 6 and above.[10] If ICON is to substitute IOTN in United Kingdom, it needs to include those cases recognized by IOTN in need for orthodontic treatment. But even then, it has been shown that a cut-off point of 43 in ICON provides lower treatment threshold.[10] This might lead to extra costs for orthodontic treatment.[6] In our country, insurance does not cover the costs of orthodontic treatment. Moreover, this notion that “every patient who is willing for orthodontic treatment and can afford it is considered as a potentially good candidate for orthodontic treatment” might have biased the examiners over the years of clinical practice.

The main shortcoming of the ICON index was the high weighting of the AC index. This item has the highest weighting in the ICON index (a weighting of 7) and therefore, it is expected that the results of the ICON index are heavily dependent on the AC component of IOTN and how it is obtained.[10] It has been concluded that whether the examiner was calibrated in measuring the ICON index or not could highly affect the results and it was mainly based on the AC component.[4] AC has not only a high weighting, but also is the most difficult to learn between different components that are considered in the ICON as opposed to easier components in this index such as the presence of cross bite, open bite or crowding. In fact, AC is reported to have a low validity.[13, 16] The incorporation of AC into the ICON index, thus, necessitates the calibration of orthodontists before any epidemiologic studies are to be carried out. Even then, the calibration process is not guaranteed, since it is highly biased by the experience, personal preferences, and abilities to learn AC scoring system.[17]

As for the complexity of the cases for orthodontic treatment, the ROC curve showed the proposed borders by Daniels and Richmond not to be applicable to our society (Table 4). The examiners also failed to differentiate between grade 3 (moderate) and 4 (difficult) of the complexity component and define a definite range for each. It has been suggested that having a high percentage of the cases in the borderline group (grade 3 of DHC) would influence the accuracy of results by making the decision more difficult for the examiners.[6] In our study, 31% of the cases were in the extreme ranges of the DHC index (either being easy or very difficult) and only 23% in the borderline group. But even though, no definite ranges could be introduced between moderate and difficult cases. On the other hand, the inter-examiner agreement in the present study was moderate and higher than the levels of agreement in Savastano *et al.* who reported an inter-examiner reliability of 0.33.[5] Despite this fact, they considered this value as reliable and this component of the index as validated. The complexity of treatment seems to be highly variable and dependant on the idea of the examiner. Further studies including a larger group of experts is required to set new ranges for assessing the complexity of treatment in our society since it seems that the one suggested by Daniels and Richmond may not be applicable for our country.[3]

## Conclusion

In terms of orthodontic treatment need, there was substantial agreement between the gold standard and the ICON index and thus the ICON index seems to be a viable candidate to substitute the IOTN index. ICON is a valid index in assessing the need for orthodontic treatment in Iran when the cut-off point is adjusted to 35. When the cut-off point was adjusted to 35 in lieu of 43, the highest level of sensitivity and specificity can be obtained for our society. The complexity component of the index was not validated in our country. Therefore, thus far, there is not a validated index for assessing the complexity of orthodontic treatment in our society. 
